# Timing Embryo Segmentation: Dynamics and Regulatory Mechanisms of the Vertebrate Segmentation Clock

**DOI:** 10.1155/2014/718683

**Published:** 2014-05-07

**Authors:** Tatiana P. Resende, Raquel P. Andrade, Isabel Palmeirim

**Affiliations:** ^1^Life and Health Sciences Research Institute (ICVS), School of Health Sciences, Campus de Gualtar, University of Minho, 4710-057 Braga, Portugal; ^2^ICVS/3B's Government Associate Laboratory, Guimarães, Braga, Portugal; ^3^Instituto de Engenharia Biomédica (INEB), Universidade do Porto, Rua do Campo Alegre 823, 4150-180 Porto, Portugal; ^4^Regenerative Medicine Program, Department of Biomedical Sciences and Medicine, University of Algarve, Campus de Gambelas, 8005-139 Faro, Portugal; ^5^Institute for Biotechnology and Bioengineering (IBB), Centre for Molecular and Structural Biomedicine, University of Algarve, Campus de Gambelas, 8005-139 Faro, Portugal

## Abstract

All vertebrate species present a segmented body, easily observed in the vertebrate column and its associated components, which provides a high degree of motility to the adult body and efficient protection of the internal organs. The sequential formation of the segmented precursors of the vertebral column during embryonic development, the somites, is governed by an oscillating genetic network, the somitogenesis molecular clock. Herein, we provide an overview of the molecular clock operating during somite formation and its underlying molecular regulatory mechanisms. Human congenital vertebral malformations have been associated with perturbations in these oscillatory mechanisms. Thus, a better comprehension of the molecular mechanisms regulating somite formation is required in order to fully understand the origin of human skeletal malformations.

## 1. Early Events in Vertebrate Development

**Figure 1:**
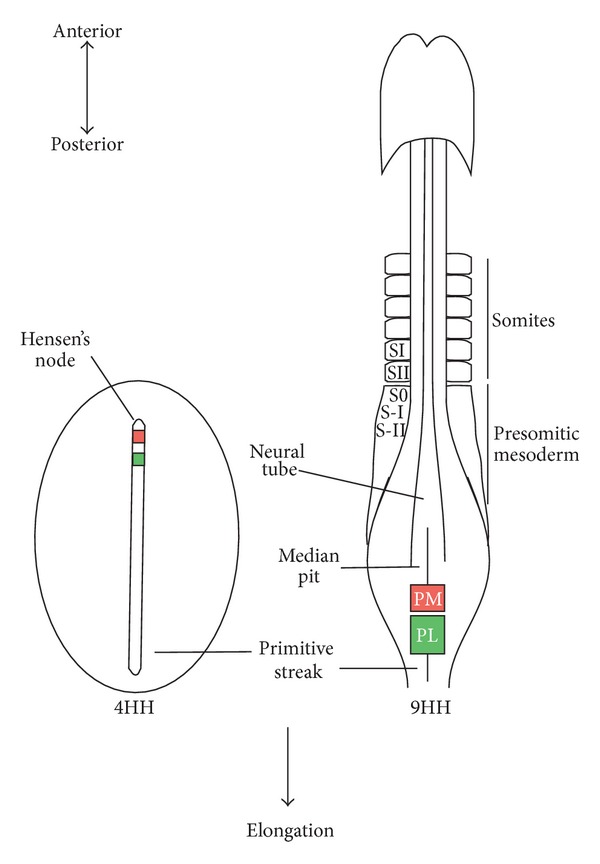
Segmentation of the chick embryo. Schematic representation of two distinct development stages of the chick embryo (dorsal view, 4HH and 9HH [2]). Hensen's node organizer is visible at the anterior tip of the fully extended primitive streak at stage 4HH. As Hensen's node regresses caudally, anterior structures are laid down as gastrulation occurs in the posterior part of the embryo. Consequently, embryonic structures are formed in a head-to-tail fashion and a clear anterior-posterior gradient of differentiation is observed, as depicted in the 9HH embryo. At this stage, a pair of somites is formed from the anterior portion of the presomitic mesoderm flanking the axial neural tube and notochord every 90 minutes. Somites are numbered according to their maturity along the A-P axis: the forming somite is designated S0, and the following presumptive ones are denoted in negative numerals (S-I, S-II, etc.) whereas segmented somites are represented with positive numerals, with SI being the most recently formed somite [30, 31]. Concomitant with somitogenesis, the embryo elongates due to the continuous contribution of new cells from the tail-bud region until the final number of somites is reached. The localization of the prospective PSM territories, medial (PM, pink) and lateral (PL, green), is indicated in both stages.

Body segmentation can be detected early in development through the formation of repeated segments, the somites, along the anterior-posterior (A-P) body axis. Somites are blocks of cells formed from the anterior end of the mesenchymal presomitic mesoderm (PSM) and have a key role in the subsequent patterning of the body giving rise to all segmented structures in the adult body, such as vertebrae, intervertebral disks and ribs, the dermis of the back, and body skeletal muscles, except those of the head. PSM is formed during gastrulation, in which extensive cellular rearrangements take place to form the three embryonic germinative layers: ectoderm, mesoderm, and endoderm. Gastrulation begins with the formation of the primitive streak (PS), first identified as a posterior thickening of the epiblast. Distinct models have been proposed to explain the specific cellular mechanism underlying PS formation (reviewed in [1]). As epiblast cells ingress and adopt distinct fates, the PS elongates towards the future anterior region and the body axes are defined. In the chick embryo, the fully extended PS corresponds to the developmental stage 4 of Hamburger and Hamilton (HH) [2], where Hensen's node (HN), which constitutes the embryonic organizer, can be detected as a cellular thickening at the PS tip (Figure 1). Cells migrating through the PS undergo an epithelial-to-mesenchymal transition and become organized in a head-to-tail fashion: the earliest cells to ingress will be positioned more anteriorly than cells migrating later in development. As a consequence, avian and mammalian embryos display a clear A-P gradient of developmental maturity: as cell ingression occurs, the HN regresses to a more posterior position, laying down the axial and anterior structures while gastrulation is still taking place at the embryo tail. In the chick embryo, PS regression is completed around the 16-somite stage. From 16 to 20 somite stages, new mesodermal cells contributing to more caudal fates arise from the tail-bud, a mass of highly packed undifferentiated cells which corresponds to a functional remnant of the primitive streak [3–5]. Lineage-tracing approaches have localized the PSM precursor cells (P-PSM) in the early chick embryo: bilateral to the midline in the epiblast (3HH), in the PS anterior region at stage 4HH, and later in the tail-bud [6–12] (Figure 1). The prospective PSM territory exhibits stem cell behaviour in both chick and mouse embryos, contributing to the formation of all axial levels of the PSM [12–15]. The mechanisms underlying HN regression and body extension are not completely understood. Regarding regulation of cell movement, a detailed analysis at stage 4HH has shown that cell ingression occurs in response to chemotactic signals belonging to the fibroblast growth factor (Fgf) family: cells are attracted to Fgf4 present in the anterior-most portion of the streak and repealed by Fgf8 from the posterior region [16].

These early morphogenetic processes in vertebrate body formation are crucial for the correct organization of the adult body and have been thoroughly studied. We now know that the final position of PSM cells in the embryo depends both on the time at which they are produced (which specifies their A-P position) and on their location along the primitive streak A-P axis, which determines their position along the medial-lateral (M-L) embryo axis (Figure 1). However, P-PSM cells in the primitive streak are not completely committed and their fate can be changed if grafted into a different A-P position [17, 18]. Interestingly, this plasticity is higher in “younger” caudal mesodermal cells, reflecting the A-P gradient of developmental maturity [18].

## 2. Segmentation of the Vertebrate Body

Somites bud off in pairs at a rhythmical pace from the rostral PSM and flank the axial structures, the neural tube, and notochord (Figure 1). As described above, the embryo concomitantly elongates due to continuous cell ingression and proliferation in the tail-bud region. The total number of somites and the periodicity of their formation are species-specific parameters. A new pair of somites is formed every 30, 90, and 120 minutes in the zebrafish, chick, and mouse embryos, respectively. Slight variations in this periodicity have been observed along the development of both mouse and chick embryos. The first and last somites were shown to be formed at a faster and slower pace, respectively, than the considered 120 minutes in mouse [19] and formation of the last pairs of somites was shown to require ~150 minutes in the chick [20].

During somitogenesis, posterior PSM is continuously replenished by cells that are progressively displaced anteriorly until somite integration. Cell tracking showed that during this process cells disperse along the PSM and often change neighbors, slowing down when distanced four to five somites from the anterior PSM tip [21, 22]. Somite formation involves extensive cellular readjustments, namely, cell packing and polarization, when preparing for the required mesenchymal-to-epithelial transition. PSM epithelialization in the chick was shown to start from the medial-most cells that elongate and recruit neighboring ones until the somite pulls apart from the PSM through a ball-and-socket mechanism [22, 23]. After somite formation, the outer epithelial cells are still able to move and change places with the luminal mesenchymal cells [23]. Somite cell compaction is accompanied by arrangements in the extracellular matrix, which has also been implicated in somite formation. In fibronectin null mouse embryos, even though paraxial mesoderm is formed, no morphological distinguishable somites are produced [24–26]. In fact, for proper somitogenesis to occur, the PSM needs to be surrounded by an intact fibronectin matrix [23, 27, 28]. Previous reports have shown that morphological somite formation requires the overlying ectoderm [29], which functions as a source of fibronectin [28]. As somites are formed and occupy more rostral positions in the embryo, A-P somitic cells compartmentalization occurs. The resegmentation process that takes place during somite maturation is crucial to impose a segmented nature on the formation of the subsequently formed structures, the skeletal muscle, vertebral elements, and blood vessels.

The striking periodic formation of somites has intrigued researchers for many years and several experiments have been made to challenge the PSM capacity to segment. However, ablations at distinct A-P levels and heterotopic or orthotopic transplantations even after inversion of the tissue A-P orientation did not disturb the original sequence or timing of somite formation [41, 42]. This indicates that PSM cells have an intrinsic ability to segment, which was probably acquired at the time of migration through the PS [43]. Several other studies have analyzed the role of PSM surrounding tissues/structures in somite formation. Chick explants differently delimited to include either the notochord, neural tube, or both led investigators to consider somite formation independent of axial structures [44, 45]. Later, these same authors reported that following quail PSM graft into a chick embryo, the inserted tissue progressively adjusted intersomitic boundaries location to that of the host contralateral part [46], suggesting that the midline structures might be controlling somite formation.

## 3. The Clock and Wavefront Model

Several models have been proposed to explain the remarkable timely regulation of somite formation, including the cell cycle model, Meinhardt's model, and the clock and wavefront model (reviewed in [47]). The prevailing dynamic model for somitogenesis, however, is the clock and wavefront mechanism. The theoretical formulation of this model proposed the existence of two independent phenomena accounting for periodic somite formation [48]: an intrinsic biochemical oscillator, a clock, by which cells oscillate synchronously between a permissive and a nonpermissive state of somite formation and a maturation front traveling along the embryonic A-P axis, moving posteriorly in concert with the A-P differentiation gradient of the embryo, the wavefront. For a somite to be formed, a group of PSM cells in the permissive state of the clock must be reached by the wavefront of differentiation. This model was proposed following the observation that a frog blastula with reduced cell number forms a smaller embryo with the normal number of somites [48]. Remarkably, experimental data obtained to date support both assumptions of the clock and wavefront model.

**Figure 2:**
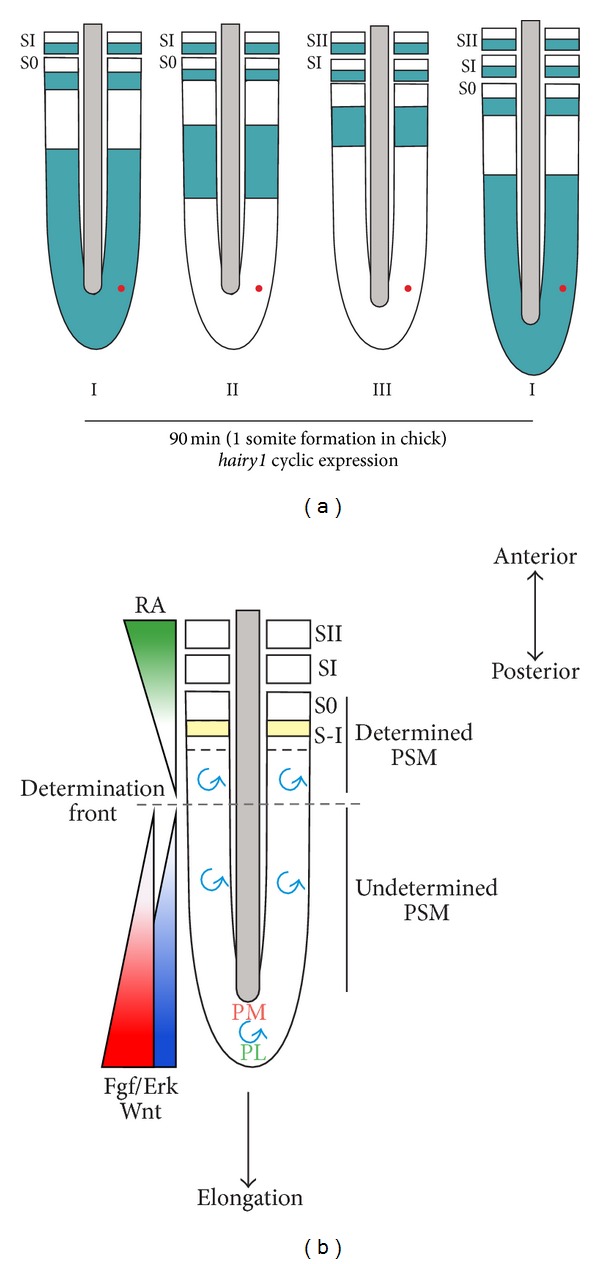
The molecular clock and wavefront. (a) Representation of the segmentation clock, visualized as distinct phases of expression (I, II, and III) of the chick oscillating gene* hairy1* [32].* Hairy1* mRNA transcriptional oscillations are propagated as posterior-to-anterior kinematic waves that sweep the PSM and culminate with the formation of a new pair of somites (SI) reiterated every 90 min in the chick. During each cycle, individual cells (represented as a red dot in the PSM) periodically turn on and off gene expression. (b) Integration of the signalling activities in the PSM regulating somite formation. Molecular clock oscillations (blue spiral) take place in the somitic precursor cells in the tail-bud region and along the entire PSM. Opposing gradients of Fgf (red), Wnt (purple), and retinoic acid (RA, green) position the determination front (dashed line) in the PSM [33–36]. High Fgf levels protect PSM posterior cells from precocious differentiation by inhibiting* raldh2* expression, thus maintaining it in an undifferentiated state (undetermined PSM). The PSM tissue above the determination front is considered to be determined and contains three to four presumptive somites [33]. Confrontation between the molecular clock oscillations and the determination front is required to define segment formation by inducing* mesp2* in the anterior PSM (yellow) [37, 38]. High Fgf/Wnt levels in the posterior PSM repress* mesp2* expression [39, 40], which is activated only when Fgf/Wnt levels drop below a threshold. S0 represents the forming somite and SI and SII represent the two most recent formed somites. PM and PL represent prospective medial and lateral PSM, respectively.

The existence of an intrinsic oscillator associated with PSM segmentation was first recognized in the chick embryo. The mRNA coding for the bHLH transcription repressor* hairy1* of the hairy/enhancer-of-split (Hes) family was observed to display different expression patterns in the PSM of stage-matched chick embryos [32]. The observed dynamic expression was reiterated every 90 minutes, corresponding to the time required to form a new pair of somites in the chick [32] (Figure 2(a)). These oscillations were shown to be an intrinsic PSM property, independent of cell movement: cells expressing* hairy1* are slightly out-of-phase along the PSM A-P axis generating a kinematic wave that sweeps the PSM [32]. These observations were later confirmed by real-time bioluminescence imaging of the* hes1* promoter in mouse embryos; waves of* hes1* transcriptional activation were seen to propagate along the PSM, briefly stabilizing in the anterior PSM before disappearing concomitantly with somite formation [49].

The second component postulated by the clock and wavefront model [48] was described in the chick a few years after the segmentation molecular clock. By performing PSM A-P inversions of one-somite length, the authors identified a region that when manipulated led to abnormal A-P somite segregation, indicating that segmental determination took place [33].* Fgf8* mRNA, expressed in the caudal portion of the PSM as a P-A gradient [33, 50], was shown to correlate with determination of front caudal regression and to be determinant for proper somite size (Figure 2(b)) [33]. Displacement of* Fgf8* gradient limit to a more rostral or caudal position led to smaller or bigger somites, respectively [33, 34, 39]. The caudal PSM* fgf8* gradient does not correspond to active transcription but rather to mRNA decay;* fgf8* transcripts are produced in the tail-bud and inherited by their descendants where the mRNA progressively decays, thus generating a gradient [34]. A gradient of Wnt/*β*-catenin signalling along the PSM, also implicated in PSM differentiation and determination of front positioning, was further described [35] (Figure 2(b)). Disruption of *β*-catenin cytoplasmatic-to-nuclear graded A-P expression had a similar effect as* Fgf8* gradient alteration, leading to extended immature PSM with no somites being formed [40, 51]. The front of determination is further refined by an antagonizing A-P gradient of retinoic acid (RA), detected by* raldh2* expression [36].

But how do the opposing gradients of Fgf/Wnt and RA regulate cell differentiation and the position of the future somite boundary? The transition from Fgf/Wnt to RA signalling constitutes a differentiation switch in the extending body axis (Figure 2(b)). High Fgf levels maintain the caudal PSM in an undifferentiated state [33], protecting the tail-bud stem cell zone from precocious differentiation by inhibiting* raldh2* expression [36]. The same authors have shown that caudal Fgf induces expression of* wnt8c*, responsible for promoting RA activity when* fgf8* levels decline as cells progress through the PSM [52]. Once cells reach the anterior PSM, the oscillatory activity and graded expression need to be converted into a cell fate change, in which somite-forming units are specified. This is accompanied by molecular changes, one of them being the periodic activation of* mesp2* expression in the anterior PSM, essential to define the future somite boundary position [37, 38].* Mesp2* has been proposed to arrest Notch oscillations leading to somite boundary formation between Notch activated and inactivated domains [37]. High Fgf and Wnt levels in the posterior PSM repress* mesp2* expression [39, 40], dictating that gene activation occurs only when Fgf/Wnt levels drop below a determined threshold. Additionally, a decrease in *β*-catenin protein's nuclear levels in the anterior PSM seems to be required for the cessation of oscillations and formation of morphological somites [51]. In *β*-catenin gain-of-function mutants no somites are formed due to the increased *β*-catenin nuclear levels and even though clock oscillations continued there was no regression of the oscillatory domain, leading to the formation of several* lfng* stripes [51]. Taken together, these results suggest that high Wnt levels in the posterior PSM provides a permissive environment for cyclic expression and that the arrest of the clock oscillations in the anterior region requires downregulation of Wnt signalling. Wnt pathway thus seems to function as a mediator of Fgf-RA inhibition, regulating the timing of PSM cells differentiation (Figure 2(b)). Interestingly, a similar relationship between these pathways has been observed in limb proximal-distal development where Fgf and Wnt pathways promote distal outgrowth while RA has an opposing proximalizing role (reviewed in [53]). This is probably a fundamental and conserved molecular mechanism that regulates differentiation progression during the development of segmented structures.

## 4. The Somitogenesis Molecular Clock

After the identification of* hairy1* cyclic expression in the chick PSM, many other genes were described to have a similar oscillatory behaviour. These are referred to as cyclic genes and constitute the somitogenesis molecular clock, suggesting that periodic somite formation is controlled by a tightly regulated genetic network. Many of the cyclic genes identified to date belong to the Notch signalling pathway (Summarized in Table 1). Among these are the* hes* genes, transcriptional repressors downstream targets of the Notch pathway. These include* hairy1*,* hairy2,* and* hey2* genes in chick [32, 54, 55];* hes1*,* hes5*,* hes7*, and* hey2* in mouse [54–57]; and* her1* and* her7* in zebrafish (reviewed in [58]). Furthermore, the Notch-modifying glycosyltransferase enzyme* lunatic fringe* (*lfng*) oscillates in the PSM of both mouse and chick embryos [59–61] and* deltaC*, a notch ligand, depicts a cyclic behaviour in zebrafish [62]. Microarray analysis of zebrafish, chick, and mouse PSM transcriptome allowed the identification of several other genes with similar dynamic expression, belonging to the Fgf and Wnt pathways (Summarized in Table 1) [63, 64]. In the mouse Notch and Fgf genes oscillate in phase with each other whereas genes belonging to the Wnt pathway are out-of-phase with Notch-Fgf [63, 64]. This was however not so evident for chick and zebrafish embryos. Several studies have tried to establish a hierarchy between the three signalling mechanisms operating in somitogenesis regulation by analysing the molecular interactions between them (Summarized in Table 2). However, it has not been easy to pinpoint a regulation chain or to identify putative molecules making the bridge between the pathways. This only shows that, due to the developmental importance of somite formation, this process is extremely well regulated. To ensure the robustness of somitogenesis, these signalling cascades are probably working synergistically, creating a complex and highly efficient signalling network.

It is now evident that the molecular events underlying somitogenesis are highly conserved among vertebrates, since periodic gene transcription has been described in multiple animal models, mouse, zebrafish, frog, and medaka (reviewed in [76]). This rhythmic expression begins as early as gastrulation, where the clock genes* hairy1*,* hairy2,* and* lfng* are dynamically expressed in the PSM precursor's cells [7, 77]. These observations suggest that the segmentation clock may be involved in defining cell fate: the number of oscillations experienced by a cell may correlate with its future localization level along the A-P body axis. A similar behaviour was observed in limb chondrogenic precursor cells, where the* hairy2* gene was shown to present an oscillatory period of 6 hours, underlying the formation of limb skeletal structures [78]. This suggests that gene oscillations with distinct periodicity may be involved in regulating the formation of different embryonic structures and can be a widespread mechanism experienced by many cell and tissue types in the vertebrate body. In fact, work performed in Kageyama's lab reported the oscillation of* hes1* gene in cultured cell lines such as myoblasts, fibroblasts, neuroblastoma, and teratocarcinoma cells [79]. Furthermore, dynamic expression of * hes* genes has also been described in mouse neural progenitors [80]. Cyclic oscillations of* hes* genes in stem cells have been correlated with the maintenance of pluripotency and regulation of binary cell fate decisions, thereby generating cell type diversity (reviewed in [80–82]). Although a human segmentation molecular clock has not yet been demonstrated experimentally due to the inability to examine early developmental events in human embryos, the phenotypes associated with human vertebral malformations are very similar to the mutation phenotypes observed in mice models (reviewed in [83]). In human, it is considered that somitogenesis occurs between 20 and 35 days after conception and the formation of each pair of somites takes around 4–6 hours (reviewed in [83]). Promising results were obtained by performing microarray analysis in a human mesenchymal stem cell population derived from umbilical cord blood [84]. Quantitative PCR analysis confirmed that* hes1* gene oscillates in these cells with a 5-hour periodicity. Hence, the data obtained so far strongly suggests that an oscillatory mechanism underlying axial skeleton formation may also be a conserved trait in humans.

## 5. Molecular Clock Regulatory Mechanisms

Theoretical predictions and experimental observations indicate that embryo segmentation is a precisely regulated mechanism that requires timely gene expression oscillations. When the expression of a given gene or protein is either knocked down or constitutively activated, phenotypic defects are depicted, indicating that the consecutive transitions between maximal and minimal molecular values observed during oscillations are necessary for proper development. In fact, over- or sustained expression of a given clock gene induces segmentation defects and consequent severe skeletal malformations such as disorganized and fused vertebrae and ribs. For example, mutant mice for* lfng* or* hes7* present short tail and trunks, the vertebrae formed are shorter in length, and the ribs are usually fused and bifurcated [85–87]. Moreover, sustained expression of* lfng* disrupts molecular oscillations and normal somitogenesis, creating offspring with shorter and kinked tails and fused vertebrae and ribs [85, 88]. Noticeably, this oscillatory behavior also seems to be implicated in stem cell fate determination: cyclic* hes1* expression in embryonic stem cells seems to contribute to the heterogeneous response of the cells and low or high* hes1* levels are determinant to specify cells to either a neuronal or mesodermal fate, respectively [82].

The description of several Notch-independent genes exhibiting an oscillatory pattern in the PSM [63, 64] has brought new insight into the molecular orchestration of the oscillations. These synchronous oscillations have intrigued developmental biologists since they were first described and in an effort to achieve a better understanding of this behaviour, several attempts have been made to interfere with this robustly regulated mechanism. Mathematical modeling predicts that alterations in protein synthesis and degradation rates should change the oscillations period [79, 94] and this has been confirmed experimentally (Summarized in Table 3). In zebrafish, disruption of Delta-Notch coupling induced an increase in the somitogenesis period and somite size until the complete loss of synchrony, when no more somites were formed [93]. Regarding the chick embryo, explant incubation in the presence of the CKI-7 Wnt inhibitor led to the formation of one less somite boundary than the control and slowed down the pace of the clock from 90 min to 115–120 min [89]. A similar effect on the oscillatory period was observed in the mouse, when inhibiting Wnt pathway after explant culture with CKI-7 [89]. Contrarily to the results obtained in zebrafish, in this case the A-P somite length was not reported to be longer. A more dramatic effect in chick somitogenesis clock was reported following surgical separation of the notochord from the PSM, resulting in slower somite formation and altered cyclic gene expression on the notochord-ablated side [92]. Inhibition of Sonic Hedgehog (Shh) signalling rendered similar results and the observed phenotype was restored by exogenous supplementation of Shh or RA. Shh was for the first time implicated in regulating both components of the clock and wavefront model: oscillations of the segmentation clock genes* hairy2* and* lfng* and expression of the determination of front defining genes,* fgf8* and* raldh2 *[92]. This concerted action of Shh may explain why even though the somitogenesis clock was delayed almost 3 hours no alterations in A-P somite length were detected [92]. As in zebrafish, alterations in the clock period were observed after disrupting Notch signalling in the mouse [90]. When using mutant mice for Notch-regulated ankyrin repeat protein (Nrarp), Notch activity was enhanced and an extension of 5 minutes in segmentation period was observed, decreasing the number of somites and resultant vertebrae formed. Contrastingly, Notch inhibition led to a shorter period of segmentation and consequent increase in somite number and vertebrae formed. As in the chick, no changes in somite size were observed [90]. This result could be explained by a concomitant alteration in the wavefront position, as was shown after PSM separation from the notochord [92]. A reduction in the number of introns within the* hes7* gene, however, led to accelerated oscillations associated with decreased somite length [91]. So far, clock period acceleration was only attained when disturbing Notch pathway in the mouse embryo [90, 91]. In the chick experiments, clock acceleration was not achieved even after overactivating Wnt signalling pathway [95] or when greatly increasing the amounts of exogenous Shh, RA, or both [92]. Thus, even though important breakthroughs have been made over the last years regarding our understanding of the embryonic molecular mechanism regulating the size, number, and identity of the segmented structures, many questions remain unanswered.

Several aspects of the molecular clock regulation mechanisms are not yet understood, especially regarding the level of crosstalk between the different pathways regulating the oscillatory behaviour. Presently, the genes that are driving oscillations or only permitting them remain largely unknown. Furthermore, it is thought that the Notch, Wnt, and Fgf oscillators need to be entrained by a so-called pacemaker to ensure that oscillations occur with the correct periodicity. The nature of this pacemaker overseeing the molecular clock oscillations and how this control is made has not yet been clarified. The past years have provided important progress in the understanding of vertebrate embryo segmentation. A big step forward was taken with the establishment of the real-time bioluminescence imaging technique in mouse embryos, which constitutes a powerful tool to further study the mechanics of clock oscillation and regulation* in vivo* [49]. However, the knowledge and understanding of the molecular events underlying it are still limited.

## 6. Cell Synchronization and the Segmentation Clock

As discussed above, generation of robust waves of gene expression requires a tight molecular control within the PSM. This is achieved by regulated single cell oscillations and cell-to-cell synchronization. At the time of the first description of the segmentation clock, it was demonstrated that the observed dynamic expression does not result from a wave travelling along the PSM axis but rather consists in a kinematic wave: gene expression in each PSM cell is slightly out-of-phase relative to that of the neighbouring cell [29, 49]. Several studies have focused on analysing cell autonomous and cell-cell synchronized oscillations. Studies in the chick embryo have shown that isolated pieces of PSM were able to maintain timely oscillations up to 6-hour incubation [96]. However, when posterior PSM cells were dissociated and separately cultured they rapidly felt out of synchrony [96]. Real-time bioluminescence imaging of mouse* hes1* dynamics for 15 hours showed that dissected PSM fragments are capable of maintaining stable expression but gradually become out-of-phase [49].* Hes1* cycles in dissociated PSM greatly varied in period and amplitude between individual cells showing that the oscillator is unstable in isolated cells [49]. Oscillations at the single cell level rely on negative feedback regulation and short lived mRNA and proteins.* Hes* genes encode nuclear proteins that act as transcriptional repressors and negatively regulate their expression via direct binding to their own promoter [97]. Thus, the oscillator period depends on the timing of transcription, translation, protein decay, and additional events such as splicing and posttranslation modifications. For example, deletion of* hes7* introns was shown to completely abolish oscillations [98]. Recently, microRNAs have been implicated in posttranscriptional regulation of oscillatory genes in the segmentation clock, controlling genetic dynamic expression [99, 100]. Inhibition of mir-125a-5p induces stabilization of chick* lfng* transcripts and subsequent loss of robust clock oscillations, associated with perturbations in somite formation and patterning [100].* Hes1* stability was shown to be regulated by miR-9, able to dampen gene oscillations when overexpressed or inhibited [99]. The authors describe a double negative feedback loop between* hes1 *and miR-9 and propose that this regulatory feedback may be responsible for termination of* hes1* oscillations and further cellular differentiation [99].

**Figure 3:**
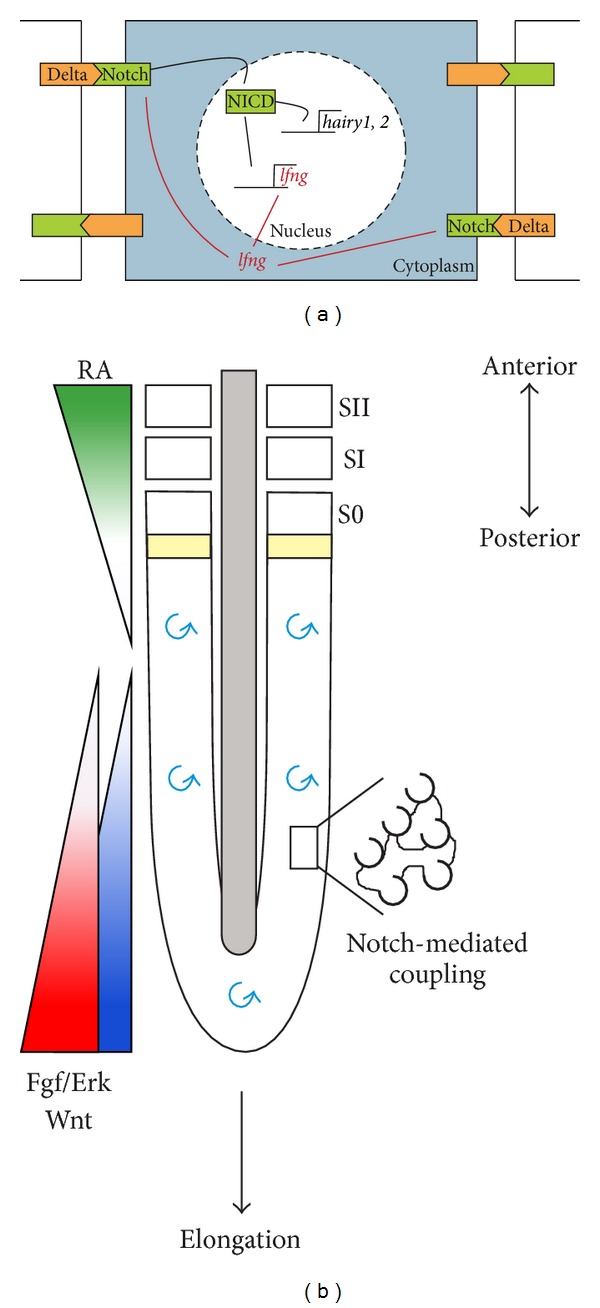
Cell-cell coupling in the somitogenesis clock. (a) Representation of the intercellular coupling achieved by Delta/Notch communication and the feedback loop underlying the generation of* lfng* oscillations in the chick embryo. The Delta ligand binds Notch receptor on adjacent cells activating the Notch signaling cascade. Notch intracellular domain (NCID) is released and translocated into the nucleus, where it activates the transcription of* hairy1*,* hairy2,* and* lfng* (black arrows). Lfng protein modifies Notch making it less sensitive to activation by Delta (red arrows). This effect is transient due to the short life of* lfng*. Oscillations are thus generated by alternation between activation of* lfng* expression and repression of Notch by* lfng* [101]. (b) Molecular clock synchronization through intercellular coupling occurs during somitogenesis: cyclic expression in the individual cell is synchronized by cell-cell coupling mediated by the Notch signaling pathway (black spiral). This intrinsic mechanism ensures robust oscillations (blue spiral) and rapid recovery following external perturbations [102]. S0 represents the forming somite and SI and SII represent the two most recent formed somites.

Individual PSM oscillations are noisy but can be synchronized at the tissue level by cell-coupling (Figure 3). Transplantation of PSM cells from a zebrafish with continuously activated Notch to a wild-type embryo caused acceleration of* her1* oscillations in adjacent cells and consequently an anterior shift of the segment positions [103]. The authors further showed that Notch attenuation resulted in variable* her1* levels from cell to cell. Thus, the global oscillation pattern in the PSM seems to be maintained by Notch-intercellular communication, as demonstrated by* in vivo* and* in silico* experiments [103]. Similarly to zebrafish, intercellular coupling in chick and mouse embryos also involves Notch signaling (Figure 3(a)). Mouse cells otherwise unsynchronized, cultured in the presence of Dll1 protein, are able to perform* hes1* mRNA and protein oscillations with a two-hour periodicity [79]. Notch-mediated intercellular coupling seems thus to be essential for synchronization of single-cell oscillations, which is crucial for molecular clock oscillations along the PSM and proper somite formation (Figures 3(a) and 3(b)). In fact, interfering with distinct components of Notch pathway leads to defects in segmentation: cyclic gene expression is disrupted and anterior somites are formed while posterior ones are disorganized with irregularly spaced boundaries (reviewed in [104, 105]). In zebrafish* deltaC* mutants, the gene is still expressed in the PSM but in an uncoordinated way: a “salt-and-pepper” pattern is observed, suggesting that cells are still oscillating individually but no longer in synchrony with their neighbors [62].

Altogether these results show that PSM cell coupling, mediated by Notch signaling, is essential to prevent cellular desynchronization and subsequent loss of segmental patterning. These experimental observations have been supported by mathematical modeling [49, 103, 106]. More recently, a theoretical model considering dynamic PSM cell rearrangement [21, 22] showed that random cell movement promotes segmentation clock synchronization and allows a faster recovery in oscillations after an external perturbation [102]. PSM seems to possess distinct intrinsic mechanisms to ensure minimal external perturbations, as recently shown [107]. The authors reported that clock oscillations and mitosis are highly coordinated in the PSM: mitotic divisions seem to occur mainly in the oscillatory “off phase” period to ensure minimal interference with molecular segmentation [107].

## 7. Final Remarks

A better comprehension of the molecular mechanisms underlying somite formation is required not only for the sake of basic developmental biology studies but also with the aim of dissecting the origin of human skeletal malformations. Several mice mutants have been produced to analyse somitogenesis gene function, which have proven to constitute good tools to understand congenital vertebral malformations in human. When the generated mutation is not lethal and it is possible to follow the development of the embryos, the most common defects are shorter trunks with fused or bifurcated vertebrae and ribs (reviewed in [108]). Similar segmentation problems are observed, for example, in patients with mutations in Notch-associated genes, which exhibit a short trunk due to multiple hemivertebrae formation accompanied by rib fusions, bifurcations, and deletions (reviewed in [83, 109]).

Even though many breakthroughs have been made over the recent years concerning the understanding of the molecular mechanisms driving proper clock oscillations and correct vertebrate development, important aspects of this intricate machinery are not entirely understood. Regulation of initiation, establishment, maintenance, and arrest of this rhythmicity has not yet been completely unravelled. Additionally, comprehension of the crosstalk and interregulation between the pathways implicated in the oscillatory behaviour has not been achieved. This is probably because the cyclic behaviour is attained by combinatorial negative autoregulation and intercellular coupling to produce robust oscillations, thus protecting embryonic development from perturbations. The focus of the developmental biology field has now been to create and implement real-time imaging techniques, which will allow studying the clock oscillation and regulation* in vivo*. Additional integration of the acquired experimental data with theoretical knowledge from mathematical modelling will also bring forward a better understanding of this complex network.

## Figures and Tables

**Table 1:** Genes belonging to the signalling pathways Notch, Wnt, or Fgf shown to have an oscillatory behaviour in mouse, chick, and zebrafish embryos.

	Mouse	Chick	Zebrafish
Notch	*hes1* [54]	*hairy1* [32]	*deltaC* [62]
	*hes5* [57]	*hairy2* [54]	*her1* [65]
	*hes7* [56]	*hey2* [55]	*her7* [66]
	*hey1* [64]	*lfng* [59, 61]	*her2/4/15* [64]
	*hey2* [55]	*nup37* [64]	*nrarp* [67]
	*lfng* [60]	*nrarp* [67]	
	*nkd1* [68]		
	*nrarp* [63]		

Wnt	*axin2* [35]	*axin2* [64]	*tbx16* [64]
	*dact1* [69]	*gpr177* [64]	
	*dkk1* [64]	*T* [64]	
	*myc* [64]		
	*sp5* [64]		
	*tnfrsf19* [64]		

Fgf	*Bcl2/11* [64]	*dusp6/2* [64]	*tbx16* [64]
	*dusp4* [70]	*fgf3* [64]	
	*dusp6* [63]	*Snail1* [64]	
	*egr1* [64]	*snail2* [71]	
	*shp2* [64]	*raf1* [64]	
	*snail1* [71]		
	*spry2* [63]		
	*spry4* [72]		

**Table 2:** Mutations in key genes belonging to the Notch, Wnt, and Fgf signaling pathways and subsequent phenotype observed in the expression of genes involved either in the molecular clock or in the wavefront of differentiation. Only mutations bringing insight into the putative cross-talk between pathways are indicated.

Pathway	Mutated gene	Effects in gene expression	References
Notch	*dll1* null	*↻axin2* *✓ fgf8*, *wnt3a *	[35]
	*hes7* null	*↻axin2, snail1, dusp6, spry1* *∅lfng, nrarp, dusp4, nkd1 *	[73] [70] [68]
	*Notch * overexpression	*↻snail2 *	[71]
	*psen1*/*psen2* null	*∅axin2*, *snail1*, *dusp6*, *spry2 *	[73]
	*Rbpjk* null	*↻spry2, dusp6, axin2, hes7, snail1* *∅lfng *	[63] [73] [71]

Wnt	Sustained *axin2 *	*∅lfng *	[35]
	*β-catenin* Gof	↑*dusp6, spry2 *	[40]
	Truncated *lef *	*∅lfng* ↓*dll1, notch *	[74]
	*wnt3a* mutant vt	*∅snail1*, *lfng, axin2, nkd1* ↓*fgf8, dll1, notch *	[71] [35] [68]

Fgf	Conditional *fgfr1 *	*∅lfng*, *hes7, spry2, axin2, snail1 *	[75] [70]
	*snail2* overexpression	*∅lfng *	[71]

*↻*: normal oscillations; *✓*: normal expression; *∅*: disrupted oscillations; ↑: increased expression; ↓: decreased expression.

**Table 3:** Perturbations of the somitogenesis molecular clock achieved in mouse, chick, or zebrafish embryos. When mentioned, alterations in clock periodic oscillations, somite formation, and wavefront gene expression are indicated.

	Perturbation	Clock	Somite	Wavefront	Reference
Mouse	⊣Wnt	↑period	n.d.	n.d.	[89]
		Δ*lfng*, *hes7, axin2*			
	*nrarp * mutant	↑period			[90]
		(95 min)	↓number (1)	*✓fgf8 *	
		*↻hes5, hes7, lfng *			
	⊣Notch	↓period			[90]
	↓*hes7* introns	↓period	↑number (1)	n.d.	[91]
		(115 min)			
			↑number (1-2)	n.d.	
			↓length		

Chick	⊣Wnt	↑period	↓number (1)	n.d.	[89]
		(115–120 min)	*✓*length		
		Δ*lfng*, *axin2 *			
	⊣Shh	↑period (~3 h)	↓number (2-3)	↓*fgf8*	[92]
		Δ*lfng*, *hairy2 *	*✓*length	↑*raldh2 *	

Zebrafish	*delta *ligands mutants	↑period	↓number (1)	n.d.	[93]
			↑length		

↑: increased; ↓: decreased; *↻*: normal oscillations; Δ: altered oscillations; *✓*: normal expression; n.d.: not done.
